# Pediatric Ocular Health and Obstructive Sleep Apnea Syndrome: A Review

**DOI:** 10.3390/pediatric15040066

**Published:** 2023-12-14

**Authors:** Marco Zaffanello, Erika Bonacci, Giorgio Piacentini, Luana Nosetti, Emilio Pedrotti

**Affiliations:** 1 Pediatric Clinic, Department of Surgery, Dentistry, Paediatrics and Gynaecology, University of Verona, 37129 Verona, Italy; giorgio.piacentini@univr.it; 2 Ophthalmology Clinic, Department of Engineering for Innovation Medicine, University of Verona, 37134 Verona, Italy; erika.bonacci@univr.it; 3 Pediatric Sleep Disorders Center, Department of Pediatrics, F. Del Ponte Hospital, Insubria University, 21100 Varese, Italy; luana.nosetti@uninsubria.it; 4 Ophthalmology Clinic, Department of Surgery, Dentistry, Paediatrics and Gynaecology, University of Verona, 37134 Verona, Italy; emilio.pedrotti@univr.it

**Keywords:** children, choroid, cornea, obstructive sleep apnea, ocular health, ophthalmology, retina, sleep-disordered breathing

## Abstract

Obstructive sleep apnea (OSA) affects neurobehavioral, cognitive, and cardiovascular aspects, particularly in children, by obstructing the upper airways during sleep. While its impact in adult ocular health is recognized, there is ongoing debate about OSA’s relevance in pediatrics. This review explores the relationship between OSA and ocular health in children, focusing on the effects and potential improvements through treatment. A systematic search found 287 articles through PubMeD/MEDLINE, Scopus, Web of Science, and ScienceDirect; 94.4% were excluded. After careful selection, six English articles were included, addressing the effects of OSA on children’s eyes. Three studies examined choroidal alterations, three explored retinal and optic nerve changes, and two analyzed ocular changes following otorhinolaryngological intervention. The immediate correlation in children is inconclusive, but age may be a contributing factor. Pediatric OSA patients exhibit corneal anomalies and increased optic nerve thickness, possibly due to intermittent hypoxia. OSA influences retinal vascular density in children, with increased density after treatment and reduced choroidal thickness in cases of adenotonsillar hypertrophy. This review emphasized OSA’s significant impact on children’s ocular health, revealing alterations in the optic nerve, choroid, retina, and cornea. While the direct correlation with the optic nerve is not always evident, OSA raises intraocular pressure and induces structural changes. Treatment holds promise, highlighting the need for regular monitoring to promptly address childhood OSA.

## 1. Introduction

Obstructive sleep apnea (OSA) is a respiratory condition characterized by the partial or complete obstruction of the upper air passages during sleep [1]. In the pediatric population, most studies report an average prevalence of 4% [2,3,4]. Sleep-disordered breathing (SDB) is more common in children aged 2 to 8 and during adolescence. The first peak of incidence is primarily associated with adenoid and tonsil hypertrophy, while the second peak is predominantly linked to obesity [5]. The prevalence is 6% in patients aged 2 to 8 years, 4.7% between 8 and 11 years, and 4.3% between 16 and 19 years [6].

The complications of SDB in the pediatric context can be categorized into major groups, namely, neurobehavioral and cognitive complications [7], growth retardation [8], and metabolic and cardiovascular issues [9,10]. Individual differences have been noted in response to hypoxia, hypercapnia, and changes in airway pressure during SDB [11]. Furthermore, alterations in patterns of proinflammatory cytokine patterns have also been observed in patients with OSA [12,13]. These inflammatory changes might have implications for the pathogenesis of the conditions and the development of numerous complications [14]. As the number of children with a heightened risk of OSA grows, a significant portion of these individuals will require ongoing medical transition monitoring into their young adulthood [15].

In adulthood, OSA is strongly correlated with various ocular diseases commonly encountered in ophthalmic practice. Some of these visual consequences are reversible, but if not adequately treated, they can also permanently threaten the patient’s vision [16,17]. Recently, it has been suggested that children with OSA might exhibit ocular disorders. Ocular comorbidities could begin in childhood with OSA, persist, and worsen in subsequent ages if not appropriately managed [18].

The existing literature highlights the correlation between OSA and ocular diseases in adults, emphasizing the risk to vision if untreated. However, comprehensive studies investigating OSA’s impact on children’s eye health are lacking. Given the significant number of young individuals at risk and the potential long-term consequences, a thorough analysis of this association is crucial. This review aims to bridge this gap by exploring how OSA may negatively affect children’s eye health, identifying limitations, and contributing to a more comprehensive understanding of the relationship between OSA and pediatric eye health.

## 2. Materials and Methods

This narrative review, incorporating a systematic literature search, aims to assess the relationship between OSA and eye health in children.

Two reviewers independently extracted data from all eligible studies. The extraction process was duplicated to minimize errors and potential biases in result interpretation. Any discrepancies were resolved by a third reviewer, ensuring accuracy and consistency in the data extraction process. Reviewers also assessed the methodology of each study, including the robustness of the study design and the validity of the results, to evaluate the overall quality of the scientific evidence.

A search was conducted using four databases: PubMeD/MEDLINE, Scopus, Web of Science (access date 25 June 2023), and ScienceDirect (access date 29 November 2023) employing specific keywords to identify relevant studies for the review.

Inclusion criteria:

Keywords included factors such as age (children, infants, adolescents), ocular diseases (eye, cornea, retina, optic nerve), and sleep disorders (sleep-disordered breathing, obstructive sleep apnea, polysomnography). These choices aimed to explore the relationship between children’s eye health and sleep disorders, tailored to the requirements of each search engine.

Exclusion criteria:

Articles in languages other than English were excluded to avoid potential language barriers. Bibliographic reviews, isolated clinical cases, case series, and letters were not considered, as they might lack the depth required for the present analysis. Studies involving adult participants (aged 18 and above) were excluded to maintain focus exclusively on the pediatric population.

Additionally, duplicate studies—those published multiple times or found in various data sources—were excluded to prevent duplications and ensure data integrity.

## 3. Results

**Figure 1:**
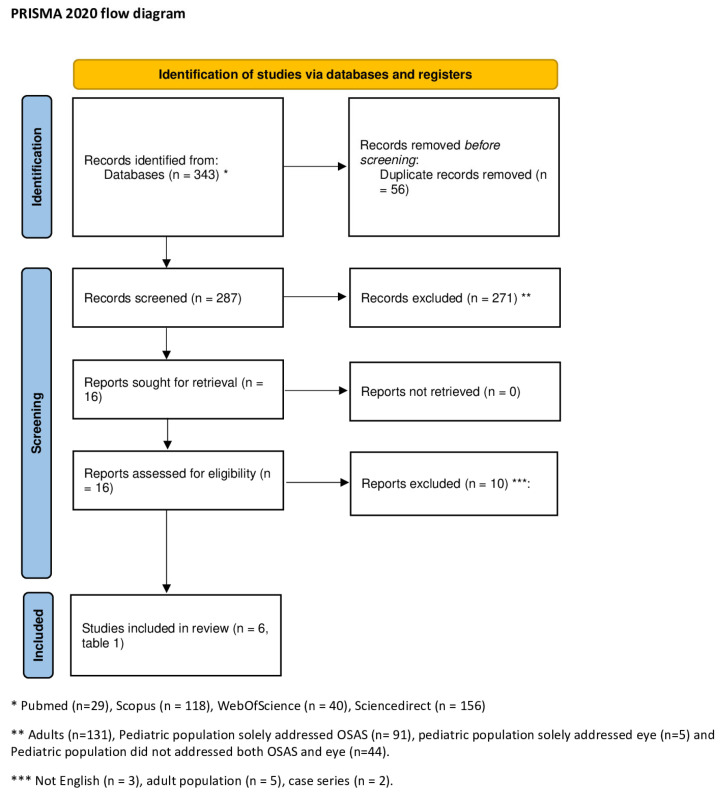
The figure displays the PRISMA 2020 flow diagram [19] for the review.

The initial search identified 343 articles, comprising 29 from PubMed, 118 from Scopus, 40 from Web of Science and 156 from ScienceDirect. Duplicate reports were subsequently excluded, removing 56 articles and leaving 29 from PubMed, 91 from Scopus, 14 from Web of Science, and 153 from ScienceDirect for further consideration (Figure 1, PRISMA). Concerning article relevance, 271 articles were excluded. Among these, 131 were associated with the adult population, while 140 pertained to the pediatric population. The latter group of papers was further categorized into three subgroups: articles excluded because they solely addressed OSA without any mention of ocular pathology (91 reports identified), articles related to ocular pathology but not OSA (5 articles placed), and articles not associated with either OSA or ocular pathology (44 articles identified). Subsequently, three articles with full text in languages other than English were excluded. Additionally, two papers were excluded due to their classification as case reports or case series, and five articles were excluded because they pertained to adulthood. Ultimately, the search yielded six relevant articles (Table 1), all related to the pediatric population and available in English.

There are three identified studies investigating retinal characteristics, the first of which was published by Cinici et al. [20]. In comparison to healthy controls, this study examined optic nerve thickness in children with OSA and adenotonsillar hypertrophy. A total of 88 patients participated in the study, but no significant correlation was found between the presence of OSA and retinal nerve fiber layer (RNFL) thickness (ranging from −0.031 to +0.016 in the right and left eyes, *p* > 0.05). However, it was noted that the age at examination might be a risk factor for developing eye issues in patients with OSA (r = +0.107, *p* < 0.05). The primary limitation of this study was the lack of polysomnography (PSG), necessitating the use of the OSA-18 survey as a substitute.

Simsek et al. [21] examined the optic nerve in 76 patients to assess differences between OSA patients and controls and identify discrepancies before and after adenotonsillectomy (A&T) in OSA patients. OSA patients exhibited higher intraocular pressure (IOP) compared to controls (*p* < 0.05), but there were no significant differences in optic nerve density. After surgery, the thickness of the superior optic nerve significantly increased in OSA patients (*p* < 0.05), correlating with the severity of the condition. The thickness of the inferior optic nerve also showed an increment in OSA patients, although it did not exhibit any significant correlation with the severity of the condition. The main limitation of the study was the lack of PSG utilization for OSA diagnosis. The results suggest that surgery can improve optic nerve thickness in OSA patients and that higher IOP may increase the risk of eye issues in moderate to severe OSA patients.

The study by Bayraktar and Şimşek [22] evaluated choroidal alterations in children with OSA. The research focused on assessing eye health in pediatric patients with OSA, specifically examining choroidal thickness. A total of 151 patients participated, including 109 with OSA and 42 controls. Choroid evaluation was conducted using optical coherence tomography (OCT), revealing a significant nasal choroidal thinning in OSA patients compared to controls (*p* < 0.05). However, the study had limitations, such as the absence of PSG and post A&T analysis, and information on disease duration. The results suggest that OSA may adversely impact children’s eye health, particularly affecting the choroid.

Ye et al. [23] examined the effect of adenoidectomy on children with OSA, utilizing optical coherence tomography angiography (OCT-A) to assess retinal perfusion. Sixty-two children with OSA were included, and a significant improvement in vascular density in the parafoveal area (*p* < 0.01) and a reduction in the foveal avascular zone were observed after the intervention (t = 4.50, *p* < 0.05) along with an increase in the deep capillary plexus (t = − 4.43, *p* < 0.05). The authors concluded that OCT-A can be considered a valuable method to evaluate the effects of adenoidectomy in pediatric OSA cases.

Ye et al. [24] assessed retinal vasculature in OSA patients compared to healthy controls, utilizing OCT-A to evaluate the choroid. A total of 132 participants were involved, including 66 cases and 66 controls. The results indicated significantly lower (*p* < 0.05) values of various vascular indices in the macular superficial/deep capillary plexus (SCP/DCP) and foveal avascular zone in the deep capillary plexus flow area zone (FAZ in DCP) in OSA patients compared to controls (*p* < 0.05). The study had limitations, such as the absence of PSG and non-measurement of arterial pressure, but it still suggests that OSA may harm retinal vasculature.

Finally, the last identified article is by Bonacci et al. [18]. Conducted in Italy on 72 patients, the study evaluated the effect of OSA on eye health. Patients were divided into two groups, with and without OSA, and were investigated with overnight respiratory polygraphy. The assessment was performed using various tools, and the results in OSA patients were found to be significantly different compared to controls in terms of corneal thickness (0.9 ± 0.5 vs. 0.6 ± 0.3, respectively; *p* = 0.02) and average retinal nerve fiber layer thickness (102.8 ± 10.5 µm vs. 98.1 ± 12.3 µm, respectively; *p* = 0.012). The study suggests that OSA may impact corneal thickness and retinal health [18].

In summary (Table 1), three studies investigating retinal characteristics [18,20,21], three examining choroids in OSA patients [22,23,24], and two on cornea [18,21] have been identified. The results indicate that OSA may harm eye health, particularly in the cornea and optic nerve. However, some studies had limitations, including the absence of PSG for OSA diagnosis [20,21,22]. Home respiratory polygraphy was used in one study [18].

Table 1 presents two prospective observational studies that evaluated ocular parameters before and after otorhinolaryngological interventions. Surgical intervention and A&T improved conditions in OSA patients [21,23]. In the first study, post A&T intervention reduced IOP and RNFL thickness [21]. In the second study, improvements in retinal vascularization were observed after adenoidectomy, with increased vascularization parameters [23].

## 4. Discussion

Out of the six identified studies, three are related to the correlation between pediatric OSA and choroidal alterations [22,23,24], and another three investigated the correlation with retinal and optic nerve changes [18,20,21]; two of the latter also considered corneal alterations [18,21]. Two studies observed ocular changes after otorhinolaryngological intervention [21,23]. However, in both studies, PSG was not utilized/stated as a diagnostic method for OSA [21,23]. These studies revealed that otorhinolaryngological interventions (such as A&T and surgery for adenoid and tonsil hypertrophy) positively impacted ocular parameters in patients with OSA, suggesting potential connections between upper airway issues and ocular changes. Five studies employed control groups to aid in comparing outcomes in patients with OSA to those without, thereby mitigating or controlling disruptive variables [18,20,21,22,24]. Only one study employed instrumental diagnosis (overnight respiratory polygraphy) of OSA [18].

In adults, OSA increases the risk of glaucoma, ischemic optic neuropathy [25,26], and floppy eyelid syndrome [17,27]. “Self-reported snoring” is associated with reduced retinal thickness and vascular density [28]. Patients with OSA exhibit reduced RNFL thickness, ganglion cell complex (GCC) thickness, and perifoveal vascular density, accompanied by retinal and optic nerve ischemic injury [29,30]. OSA may be associated with non-arteritic anterior ischemic optic neuropathy (NAION) due to endothelial damage, hypoxia, and reduced perfusion [17]. It is linked to dry eye syndrome [17,31], keratoconus [16,17], and central serous chorioretinopathy. OSA heightens the risk of retinal vein occlusion (RVO) and diabetic retinopathy [17,32], causing damage to retinal vessels [17,32]. Venkatesh et al. found increased blood flow (*p* = 0.011) and vessel density (*p* = 0.002) in the superficial capillary plexus and decreased flow (*p* = 0.003) and density (*p* < 0.001) in the deep capillary plexus among 303 high-risk OSA adult patients using the STOP-BANG questionnaire [33].

In pediatric studies of OSA, ocular changes have been observed, including optic nerve thickness, choroidal layer, retinal vascularization, and cornea. OSA in children can impact eye health, with increased retinal vascular density after treatment [23] and reduced choroidal thickness in patients with adenotonsillar hypertrophy [22]. Despite the absence of severe cases, OSA could have a negative impact, necessitating regular monitoring [24]. An immediate correlation between OSA and optic nerve thickness in children is not evident, and contradictory results have been reported. OSA raise intraocular pressure and reduces optic nerve thickness, but treatment improves the optic nerve [21]. In pediatric patients with OSA, the cornea exhibits anomalies, and optic nerve thickness increases, possibly due to intermittent hypoxia [18].

So far, the research appears to have methodological shortcomings that could impact the validity and reliability of the results. Firstly, to our knowledge, the research is based on only six studies [18,20,21,22,24], and it is possible that the sample size might not be sufficiently large to obtain representative and reliable results. The absence of PSG usage in the diagnosis could raise doubts about the accuracy of the diagnosis itself and may affect the proper identification of patients with OSA [20,21,22,23,24]. Lastly, if the patients with OSA have other medical conditions that could influence ocular parameters, these factors should be considered in the analysis [18].

In summary, the high heterogeneity among the included studies is attributed to various factors, including variable study methods, heterogeneous study populations, and differences in diagnostic techniques. This diversity makes conducting a detailed statistical analysis or meaningful subgroup analyses challenging. These factors contribute to the challenge of running a thorough statistical analysis or performing meaningful subgroup analyses in the results of the included studies.

Addressing this future challenge will involve conducting long-term studies to examine the effects of OSA on children’s eyes as they transition into young adults. It will also be crucial to plan more robust clinical trials involving a larger sample of participants, measure the severity of SDB through PSG, and carefully assess the specific eye conditions of the individuals studied. Additionally, conducting a comprehensive eye assessment is challenging in the early age group. Still, prospective studies must consider it to monitor the longitudinal evolution of ocular data and better focus on timing and treatment modalities.

## 5. Conclusions

The results of the studies indicate a potential negative impact of OSA on children’s eye health. These studies have examined various aspects, including optic nerve thickness, choroidal layer, and retinal and corneal vascularization alterations. Overall, OSA could adversely affect ocular health through multiple mechanisms. Therefore, regular monitoring by an ophthalmologist for children affected by OSA is paramount. However, it is important to note that the research conducted so far has some methodological shortcomings that could impact the validity and reliability of the results. Therefore, while the studies emphasize the significance of considering OSA as a potential influencing factor in pediatric ocular pathologies, further investigations are necessary using more accurate diagnostic methods and a larger sample size to obtain more robust and generalizable results.

## Figures and Tables

**Table 1:** Summary of studies investigating ocular effects of OSA in pediatric populations.

First Author (yr)	Country	Study Design	Patients (n.)	Age (yrs)	Ocular Area Evaluation	Methods of Ocular Assessment	Sleep-Disordered Breathing Evaluation	Aim	Result	Study Limitation
Cinici 2015 [20]	Turkey	Comparative study	88 (57 ATH, 31 controls)	8.13 ± 1.6 yrs (n. 15); 8.6 ± 2.1 yrs (n. 42); 8.59 ± 2.0 yrs (n. 31)	Retina	OCT	OSA-18 Quality of Life Survey	To assess the influence of OSA-related hypoxia on RNFL in patients with ATH	No statistical correlation between cases and controls in RNFL	No PSG used for OSA diagnosis
Simsek 2016 [21]	Turkey	Prospective comparative study	76 (42 with ATH and 34 controls)	6.62 ± 1.54 yrs (ctr n. 34); 5.98 ± 1.40 total UAO group n. 42)	Retina and cornea	Slit-lamp biomicroscopy, Perkins applanation tonometry, and OCT	Score distributions, determined by Brouillette scoring	To assess differences in retinal parameters in OSA and ATH and controls, and in OSA patients before and after A&T	Higher IOP was observed in patients with OSA. After A&T, there was a reduction in IOP and an increase in RNFL	No PSG was used for OSA diagnosis
Bayraktar 2017 [22]	Turkey	Comparative study	151 (109 OSA and 42 controls)	5.23 ± 0.75 yrs (CTR n. 42); 5.39 ± 0.90 yrs (patients n. 109)	Choroid	Slit-lamp, OCT-A, Goldmann applanation tonometry	Nocturnal pulse oximetry	To assess choroidal thickness alterations in OSA patients compared to controls	The patients exhibited choroidal thinning at 1000 µm and 1500 µm nasal compared to controls	Unknown duration of the condition; reversal of alteration after A&T not evaluated
Ye 2019 [23]	China	Prospective observational study	62	5.94 ± 1.64 yrs	Choroid	OCT	Clinical criteria	To assess retinal parameters before and after adenoidectomy in patients with OSA	Retinal vascularization showed improvements after adenoidectomy; increase in VD, VAD, VSD, and VPI	Undeclared PSG; blood pressure not assessed; not compared with controls; disease duration not considered
Ye 2021 [24]	China	Comparative study	132 (66 OSA and 66 controls)	6.23 ± 1.73 and 6.76 ± 1.89 yrs, respectively	Choroid	OCT-A	Diagnostic criteria	Compare retinal vascular characteristics in patients with OSA and controls	The VD, VAD, VSD, and VPI indices in the SCP/DCP and FAZ in DCP were lower in patients with OSA	Diagnostic criteria guidelines of pediatric OSA in otorhinolaryngology
Bonacci 2022 [18]	Italy	Observational study	72 (OSA group n. 35)	9.20 ± 3.70 yrs	Cornea and retina	Slit- lamp, Goldmann applanation tonometry, OCT	Overnight respiratory polygraphy	Investigate early cornea, macula, and optic nerve alterations in patients with OSA compared to controls	Patients with OSA exhibited higher SAI and lower TCT in the cornea and increased RNFL in the nasal quadrant.No difference in both macular volume and thickness	Reversal of alteration after OSA therapy not evaluated

Legend: A&T, adenotonsillectomy; ATH, adenotonsillar hypertrophy, DCP, deep capillary plexus; FAZ, foveal avascular zone; IOP, intraocular pressure; OCT, optical coherence tomography; OSA, obstructive sleep apnea; PSG, polysomnography; RNFL, retinal nerve fiber layer; SCP/DCP, macular superficial/deep capillary plexus; TCT, thinnest corneal thickness; SAI, surface asymmetry index; UAO, upper airway obstruction; VAD, vascular area density; VD, vascular diameter; VPI, vessel perimeter index; VSD, vascular skeleton density; yrs, years.

## Data Availability

Data sharing not applicable.
